# Identification of an Anoikis-associated LncRNA Signature to Predict the Clinical Prognosis and Immune Function of Patients with Endometrial Cancer

**DOI:** 10.7150/ijms.107243

**Published:** 2025-05-07

**Authors:** Guanxiao Chen, Ting Zhou, Xiaoyu Shen, Wan Shu, Shuyang Yu, Kejun Dong, Piotr Laudański, Klaudia Gutowska, Shuangshuang Cheng, Hongbo Wang

**Affiliations:** 1 Department of Obstetrics and Gynecology, Union Hospital, Tongji Medical College, Huazhong University of Science and Technology, Wuhan 430022, China.; 2 Clinical Research Center of Cancer Immunotherapy, Hubei, Wuhan, 430022, China.; 3 Department of Obstetrics, Gynecology and Gynecological Oncology, Medical University of Warsaw, Poland.; 4 Women's Health Research Institute, Calisia University, Kalisz, Poland.; 5 OVIklinika Infertility Center, Warsaw, Poland.

**Keywords:** anoikis, lncRNAs, endometrial cancer, prognosis

## Abstract

**Background:** Endometrial cancer is a highly heterogeneous malignancy in women with high mortality, and patients diagnosed with advanced endometrial cancer have a poor prognosis. Anoikis is a form of programmed cell death that is important for cancer development and metastasis. Long non-coding RNAs (lncRNAs) are considered critical regulators of gene expression and key players in cancer biology; however, the effects of anoikis-associated lncRNAs on the prognosis and treatment of patients with endometrial cancer remain unclear. **Methods:** Using transcriptome sequencing data and clinical information from The Cancer Genome Atlas database, we developed a novel prognostic signature for endometrial cancer based on anoikis-related lncRNAs by combining multivariate regression analysis and least absolute shrinkage and selection operator regression. The signature was validated by receiver operating characteristic (ROC) curve and Kaplan-Meier analyses. After analyzing the relationships between the seven lncRNAs in the signature and tumor progression through gene set enrichment analysis (GSEA), we further explored the differences in immune function and drug sensitivity. Additionally, to investigate the functions of these lncRNAs in the occurrence and development of endometrial cancer, we selected *CFAP58-DT* to conduct a series of *in vitro* and *in vivo* experiments to verify its partial functions. **Results:** Seven anoikis-associated lncRNAs (*CFAP58-DT, AC004585.1, AC103563.9, AL121895.2, AC004596.1, AC010761.4,* and *AC087564.1*) with prognostic value were identified for signature construction. The analysis showed excellent predictive accuracy of the signature (the largest area under the ROC curve = 0.757). GSEA indicated that these lncRNAs may regulate diverse cellular processes, including intercellular interactions, cell proliferation, differentiation, apoptosis, angiogenesis, glucose and fatty acid metabolism, immune responses, and inflammatory regulation. Furthermore, immune analysis revealed a high likelihood of immune evasion and poor immunotherapy efficacy in high-risk patients. However, there were distinct differences in the immune checkpoints and anticancer drug sensitivity between the two patient groups, which may aid in guiding treatment. Finally, our experiments showed that silencing *CFAP58-DT* significantly affected cell proliferation, promoted apoptosis, and reduced tumor malignancy. **Conclusion:** Our study highlights the significance of anoikis-associated lncRNAs in endometrial cancer progression and their potential as prognostic markers and therapeutic targets. The signature constructed using these lncRNAs may offer new avenues for endometrial cancer treatment and immunotherapy. The function of *CFAP58-DT* has been validated *in vitro* and *in vivo*, consistent with our previous analysis; however, further research into its upstream and downstream mechanisms is warranted.

## Introduction

Endometrial cancer (EC) is a prevalent malignancy of the female reproductive system that ranks sixth among the causes of cancer-related mortality in women 1. It is concerning that the annual incidence of EC is increasing 2. Although most women present with localized disease at diagnosis with a good prognosis after surgery alone, patients with distant metastasis have a poor prognosis, with a 5-year overall survival (OS) rate of 17.3% 3, 4. Currently, there are limitations to assessing the risk of metastasis and planning treatments based solely on pathology, leading to inappropriate treatment. Therefore, further exploration of EC prognosis-related markers in women is crucial for improving patient outcomes.

Cancer metastasis is a complex multistep biological process in which cancer cells invade tissues, survive in transit, and colonize organs; this process is collectively referred to as the metastatic cascade 5. As a defense mechanism against metastasis, cells typically undergo apoptosis when they lose contact with neighboring cells or the extracellular matrix (ECM). This process of cell death is referred to as “anoikis” 6. Cancer cells develop a means to evade anoikis (termed anoikis resistance), allowing them to leave the primary tumor site and survive in the absence of adhesion. This ability represents a critical step for metastasis, ensuring that circulating tumor cells can survive and eventually resume proliferation at distant sites to colonize these organs 7, 8. Therefore, a thorough analysis to identify the roles of key drivers in the occurrence of anoikis may have considerable practical implications.

Long non-coding RNAs (lncRNAs) are autonomously transcribed non-coding RNAs > 200 nucleotides in length that do not overlap with annotated coding genes 9. Recently, researchers have identified the crucial role of lncRNAs in many biological activities and the occurrence and development of cancer 10. For example, lncRNA-*Gas5* has been demonstrated to significantly enhance *PTEN* expression by inhibiting *miR-103* expression, thereby promoting cancer cell apoptosis 11. LncRNA-*Meg3* has been shown to downregulate and exhibit an anti-proliferative effect in EC by inhibiting Notch signaling 12. LncRNA-*PCGEM1* has been shown to upregulate the expression of *STAT3* by acting as a competing endogenous RNA for *miR-129-5p* 13. However, further research is required to determine the involvement of lncRNAs in EC.

To date, no studies have explored the mechanisms of anoikis-related lncRNAs in EC. In this study, we explored the role of anoikis-related lncRNAs and established a prognostic signature to predict the prognosis of patients with EC. Furthermore, we investigated its relationship with immunity and drug sensitivity, providing a basis for the personalized clinical diagnosis and treatment of EC. In addition, the role of *CFAP58-DT* in EC was partially validated.

## Methods

### Data collection

Transcriptome data were sourced from The Cancer Genome Atlas (TCGA) database (http://cancergenome.nih.gov/), which included 23 normal samples and 554 tumor samples. Furthermore, we obtained complete clinical follow-up information from the TCGA for 543 patients. We extracted and screened 638 genes related to anoikis from the GeneCards (https://www.genecards.org/) and Harmonizome (https://maayanlab.cloud/Harmonizome/) databases.

### Identification of differentially expressed (DE) anoikis-related genes (ARGs) and anoikis-related lncRNAs (ARLNCRs)

The first step was to filter the DE-ARGs using the “limma” R package with the cut-off standard as |log fold change (FC)| > 1 and a P value < 0.05. Subsequently, 4,044 ARLNCRs were identified using Pearson's correlation analysis (R > 0.4, P < 0.001). The expression levels of these lncRNAs were extracted, and 913 DE-ARLNCRs were identified (|log FC| > 1 and P < 0.05).

### Construction and validation of the prognostic signature

Samples with clinical information were randomly allocated into training and testing cohorts at a 1:1 ratio using the “caret” package. The training cohort was utilized to establish the prognostic signature, while both the testing cohort and the entire cohort were reserved for validation purposes 14, 15. Initially, we conducted a univariate Cox regression analysis to identify 68 prognosis-associated DE-ARLNCRs. Further analyses were conducted using least absolute shrinkage and selection operator (LASSO) regression and multivariate Cox regression. Ultimately, seven DE-ARLNCRs (*CFAP58-DT, AC004585.1, AC103563.9, AL121895.2, AC004596.1, AC010761.4,* and *AC087564.1*) were identified as a prognostic signature. We divided patients with EC into high- and low-risk groups based on the risk score, which was calculated as follows: risk score = Σn i=1 coef (i) × x (i), where coef (i) and x (i) represent the estimated regression coefficient and the expression value of the DE-ARLNCRs, respectively.

Risk curves, scatter plots, and heatmaps were generated using the “pheatmap” and “survival” packages to depict the risk score distribution, survival status, and expression profiles of the seven DE-ARLNCRs. Additionally, to evaluate the prognostic characteristics, Kaplan-Meier (K-M) and receiver operating characteristic (ROC) curves were plotted. Principal component analysis (PCA) was conducted using “Scatterplot3D” and the “limma” package to investigate the distribution of patients.

### Clinical evaluation of the signature

We investigated whether clinical characteristics (age, stage, and grade) and the risk score could serve as independent prognostic predictors through univariate and multivariate Cox regression analyses. Utilizing the R packages “timeROC” and “survminer,” we constructed ROC curves and calculated the area under the curve (AUC) to compare the predictive ability of different factors and to assess survival.

### Nomogram and calibration analysis

The prognostic nomogram to predict the 1, 3, and 5-year OS of patients with EC was constructed by integrating age, stage, grade, and the risk score using the “rms” R package. Calibration curves were used to visualize the results and evaluate the consistency between the predicted and actual survival. A diagonal line (45°) was recognized based on the best prediction value.

### Gene set enrichment analysis (GSEA)

GSEA was conducted using the R package “enrichplot” and “clusterProfiler” with Kyoto Encyclopedia of Genes and Genomes (KEGG) (c2.cp.kegg.v7.4. symbols.gmt) and Gene Ontology (GO) (c5.go.v7.4. symbols.gmt) analyses to explore the potential molecular mechanisms that promote EC development.

### Immune analysis and drug sensitivity calculation

The association between risk scores and immune cell populations was analyzed using CIBERSORT, TIMER, XCELL, QUANTISEQ, MCP counter, EPIC, and CIBERSORT tools. Next, the immune cells and the immune pathway infiltrating scores were explored using the “limma” and “GSVA” packages. The immune checkpoint activation between the two groups was compared using the “ggpubr” package. The “oncoPredict” and “ggplot2” R packages were used for drug sensitivity prediction.

### Cell culture

EC cell lines, including HEC-1B, HEC-1A, KLE, RL95-2, and Ishikawa, were purchased from Zhong Qiao Xin Zhou Biotechnology (Shanghai, China). Ishikawa, HEC-1B, RL-952, and KLE cells were cultured in DMEM/F12 (Gibco) supplemented with 10% fetal bovine serum (FBS) (Gibco). HEC-1A cells were cultured in McCoy's 5 A supplemented with 10% FBS. Cells were maintained at 37 °C in an incubator containing 5% CO2 and were negative for mycoplasma at the beginning and end of the experiment. The mutation status of the six driver genes in each of the cell lines used, including PTEN, TP53, PIK3CA and other genes commonly mutated in EC, are shown in Table S1.

### Quantitative real-time polymerase chain reaction (qRT-PCR)

RNA was extracted with Trizol kit and reverse transcribed to cDNA with reverse transcription kit. The qRT-PCR reaction was conducted using the real-time PCR detection system produced by Bio-Rad. The RNA expression levels were calculated by the 2^-ΔΔCT^ method using the expression of β-actin as an internal reference. The primers used in the reaction were synthesized by Sangon Biotech, and the sequences were as follows: CFAP58-DT-Forward primer 5'-GGGGTACCGGGCAGATGGAGACACCCA-3', CFAP58-DT-Reverse primer 5'-CGGGATCCTGGGTTGTTGGAAAATTTGCTCAC- 3', β-actin-Forward primer 5'-GAGAAAATCTGGCACCACACC-3', and β-actin-Reverse primer 5'-GATAGCACAGCCTGGATAGCA- 3'.

### Transfection

The shCFAP58-DT plasmid was purchased from Genomeditech (Shanghai, China). Transfection was performed using Lipo3000 (Invitrogen, USA) following the manufacturer's instructions. shCFAP58-DT: GGTACAAATAGTTGAAATA.shCFAP58-DT-negative control TTCTCCGAACGTGTCACGT.

### Cell counting kit-8 (CCK8) assay

Cells (1 × 10^4^ cells/well) were cultured in 96-well plates, and 100 µL of cell culture medium was added to each well. After incubation for 24 h, the medium in each well was replaced with fresh medium containing 10% CCK8 (ABclonal, China) and incubated for 1-2 h in the dark at a constant temperature. The optical density (OD) at 450 nm was measured.

### EdU assay

Cells (1 × 10^4^ cells/well) were plated in 96-well plates and incubated overnight. The EdU solution (Beyotime, China) was prepared at a 1:1000 concentration oof 1:1000 in the medium and added at 100 µL per well. After incubation at 37 °C for 2 h and being fixation in 4% paraformaldehyde for 15 min, the cells were incubated with 50 µL of reaction solution for 30 min in the dark. The nuclei were stained for 10 min using Hoechst. Cells were imaged using a fluorescence microscope (Olympus IX71, Tokyo, Japan).

### Calcein/propidium iodide (PI) staining

Cells (8 × 10^5^ cells/well) were plated in 6-well plates and incubated overnight. After incubating with Calcein and PI (Beyotime, China) for 30 min at 37°C in the dark, cells were observed by a fluorescence microscope.

### Apoptosis analysis

Cells (8 × 10^5^ cells/well) were plated in 6-well plates and incubated overnight. Both adherent and suspended cells were collected, followed by incubation with Annexin V-FITC and PI (Beyotime, China) for 20 min at 15-20°C in the dark. A flow cytometer (BD Biosciences, USA) was used for detection and FlowJo software was used for data analysis.

### Cell cycle analysis

Cells (8 × 10^5^ cells/well) were plated in 6-well plates and incubated overnight. After digestion, cells were washed twice with PBS, fixed in 70% ethanol at 4 ºC for 24 h. Fixed cells were resuspended in PI/RNase Staining Buffer (Beyotime, China) and incubated for 15 min at room temperature in the dark. A flow cytometer (BD Biosciences, USA) was used for detection and FlowJo software was used for data analysis.

### Colony formation assay

Cells (500 cells/well) were seeded in 6-well plates and then allowed to grow in an incubator at 37 °C for 2 weeks. Then, the cells were treated with 4% paraformaldehyde for fixation and stained with 0.1% crystal violet.

### Western blot (WB) analysis

Protein was extracted using RIPA lysis buffer. Subsequently, the bicinchoninic acid (BCA) method was employed to quantify the protein content. A polyacrylamide gel electrophoresis (PAGE) gel with a concentration of 12.5% was used for electrophoresis, and the separated proteins were then transferred onto a 0.2 μm polyvinylidene fluoride (PVDF) membrane. After incubation with skim milk for 1-2 h at room temperature and incubation with the primary antibodies overnight at 4 °C, the membranes were then incubated with the secondary antibodies for 1-2 h at room temperature. Enhanced chemiluminescence (ECL) detection reagents and a ChemiDoc imaging system were used for protein visualization.

### Tumor xenotransplantation experiments

Five-week-old female BALB/c-nu mice (Shulaibao Biotechnology, China) were maintained in a standard specific pathogen-free (SPF) environment and randomly divided into the normal control (NC) and *shCFAP58-DT* groups. Cells (1 × 10^6^) were suspended in 100 µL PBS and then injected subcutaneously into the left scapula of the mice. After 28 days, the mice were euthanized, and the tumors were excised for subsequent analysis. The morphological and structural characteristics of the tumor cells were observed by hematoxylin and eosin (H&E) staining. Differential expression of Ki67 and PCNA in tumor xenografts between the NC and *shCFAP58-DT* groups was assessed by immunohistochemistry (IHC).

### Statistical analysis

Statistical analysis was performed using Student's t-test and ANOVA with R version 4.2.3 or GraphPad Prism 9 software. Unless specified otherwise, statistical significance was established at P < 0.05.

## Results

### Identification and functional enrichment analysis of DE-ARGs

**Figure 1:**
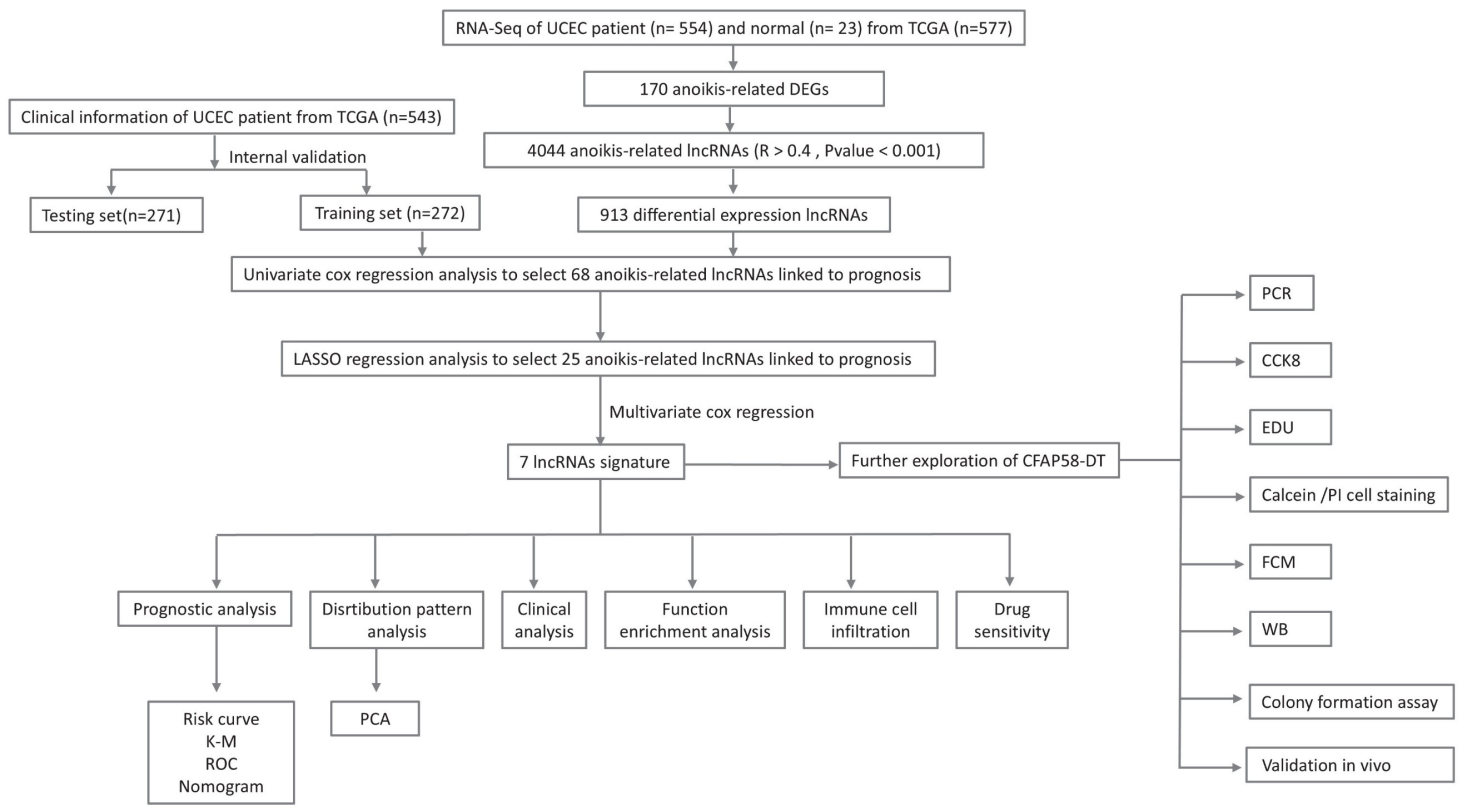
The flowchart of this study.

**Figure 2:**
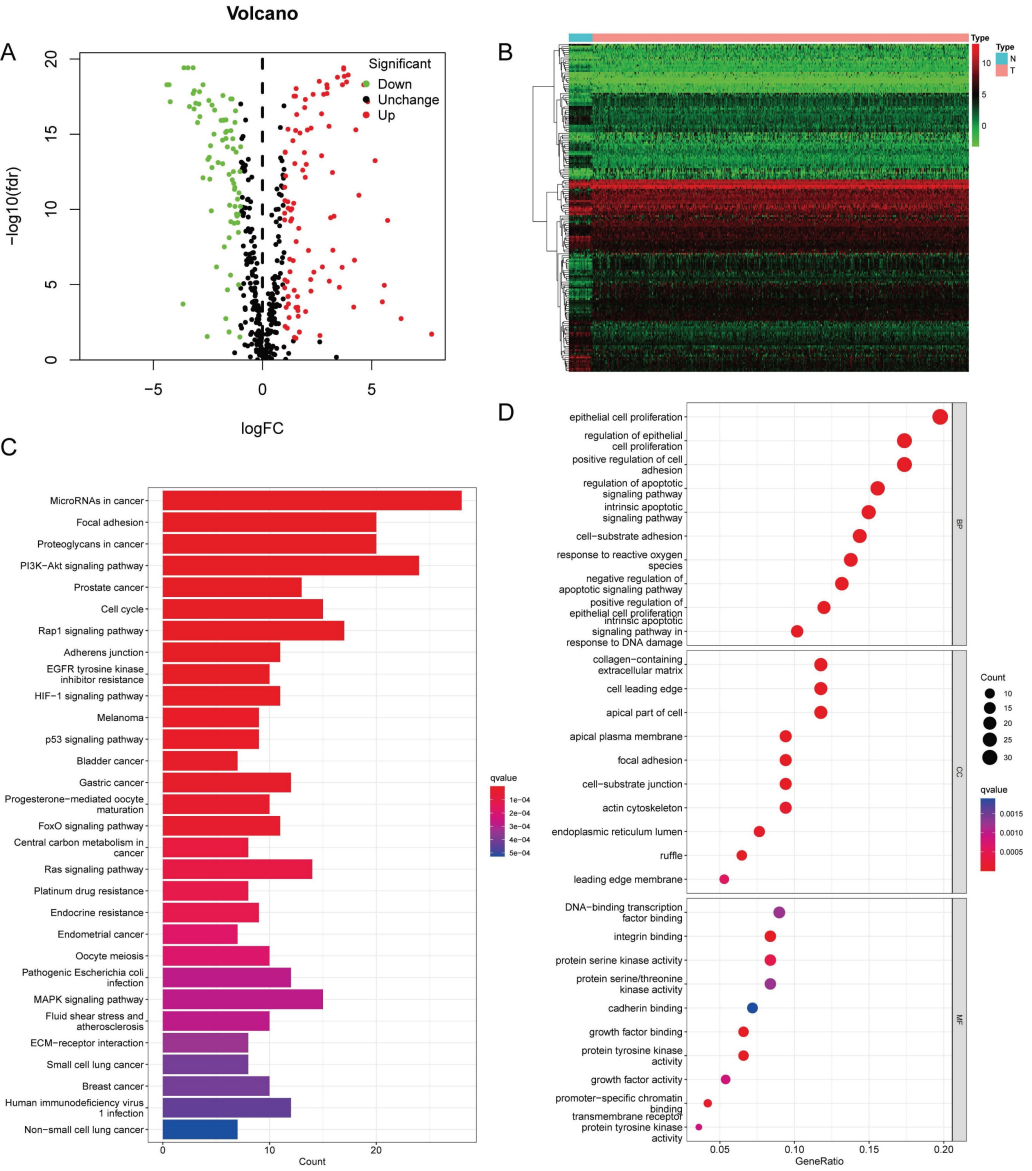
** (A)** Volcano plot identifying DE-ARGs, showing downregulated and upregulated genes. **(B)** Heatmap displaying the expression levels of DE-ARGs in each sample. **(C)** KEGG Pathway Enrichment Analysis. **(D)** GO Enrichment Analysis.

Figure 1 presents a flowchart of this study. By processing and analyzing the mRNA sequencing data downloaded from TCGA database, which encompassed 23 normal samples and 554 tumor samples, we identified 174 DE-ARGs, including 97 upregulated and 77 downregulated genes (Figure 2A, B). Subsequently, KEGG pathway analysis revealed that the DE-ARGs were enriched in pathways such as microRNAs in cancer, focal adhesion, proteoglycans in cancer, PI3K-AKT signaling pathway, prostate cancer, cell cycle, RAP1 signaling pathway, adherens junction, EGFR tyrosine kinase inhibitor resistance, and HIF-1 signaling pathway (Figure 2C). The GO analysis demonstrated that within biological processes (BP), DE-ARGs were primarily enriched in terms of epithelial cell proliferation, response to reactive oxygen species, regulation of epithelial cell proliferation, intrinsic apoptotic signaling pathway, and intrinsic apoptotic signaling pathway in response to DNA damage. In terms of cellular components (CC), DE-ARGs were mainly enriched in the collagen-containing extracellular matrix, cell leading edge, apical part of the cell, apical plasma membrane, and focal adhesion. For the molecular function (MF), DE-ARGs were predominantly enriched in DNA-binding transcription factor binding, integrin binding, protein tyrosine kinase activity, protein serine kinase activity, and protein serine/threonine kinase activity (Figure 2D).

### Construction of the DE-ARLNCR prognostic signature

**Figure 3:**
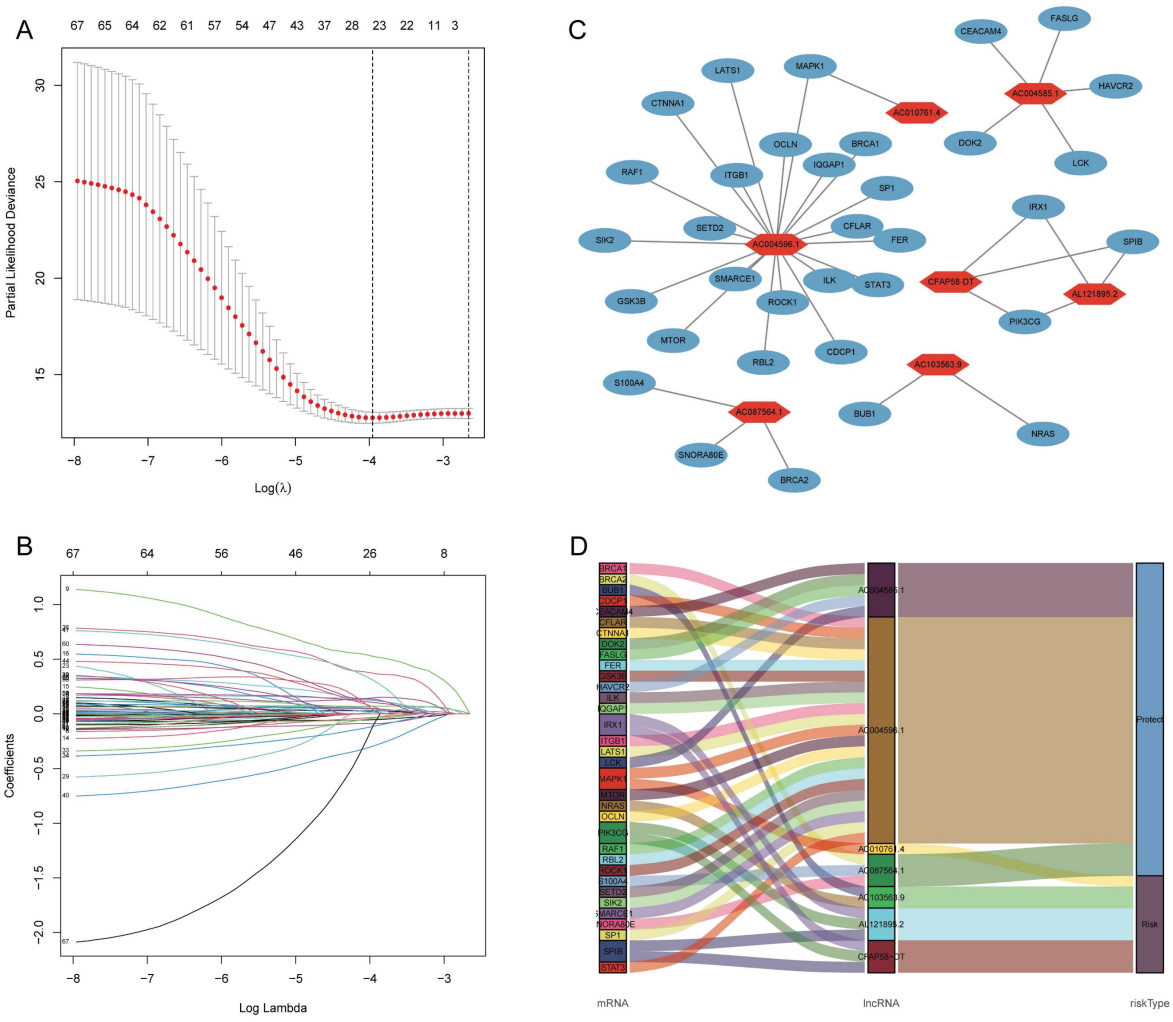
** (A, B)** LASSO regression analysis of 68 prognostic related DE-ARLNCRs. **(C, D)** The correlation between DE-ARGs and 7 DE-ARLNCRs in the signature.

Pearson's correlation analysis (R > 0.4, P < 0.001) was conducted on 174 DE-ARGs and lncRNA expression data to identify 4,044 ARLNCRs, of which 913 were defined as DE-ARLNCRs (|log FC| > 1, P < 0.05). A total of 543 patients were randomly allocated into two groups: the training cohort (n = 272) and the testing cohort (n = 271). The validation of clinical characteristics confirmed the rationality of the cohort allocation, revealing no significant differences in various clinical factors between the two groups (Table 1). Subsequently, the training cohort was utilized to develop an optimal signature for predicting prognosis based on the identified DE-ARLNCRs. Both the testing cohort and the entire patient cohort were then employed to rigorously assess the predictive accuracy and generalizability of the signature. Univariate Cox regression analysis was employed to identify 68 DE-ARLNCRs that were prognostic for patients with EC (Figure S1). To prevent overfitting, LASSO regression was employed to identify the most promising predictive biomarkers (Figure 3A, B). Furthermore, the multivariate Cox regression analysis identified seven lncRNAs crucial for the construction of the prognostic signature (Table 2). A co-expression network diagram and Sankey plot (Figure 3C, D) were used to illustrate the interactions between the ARGs and lncRNAs incorporated into the signature.

### Validation of the DE-ARLNCR prognostic signature

**Figure 4:**
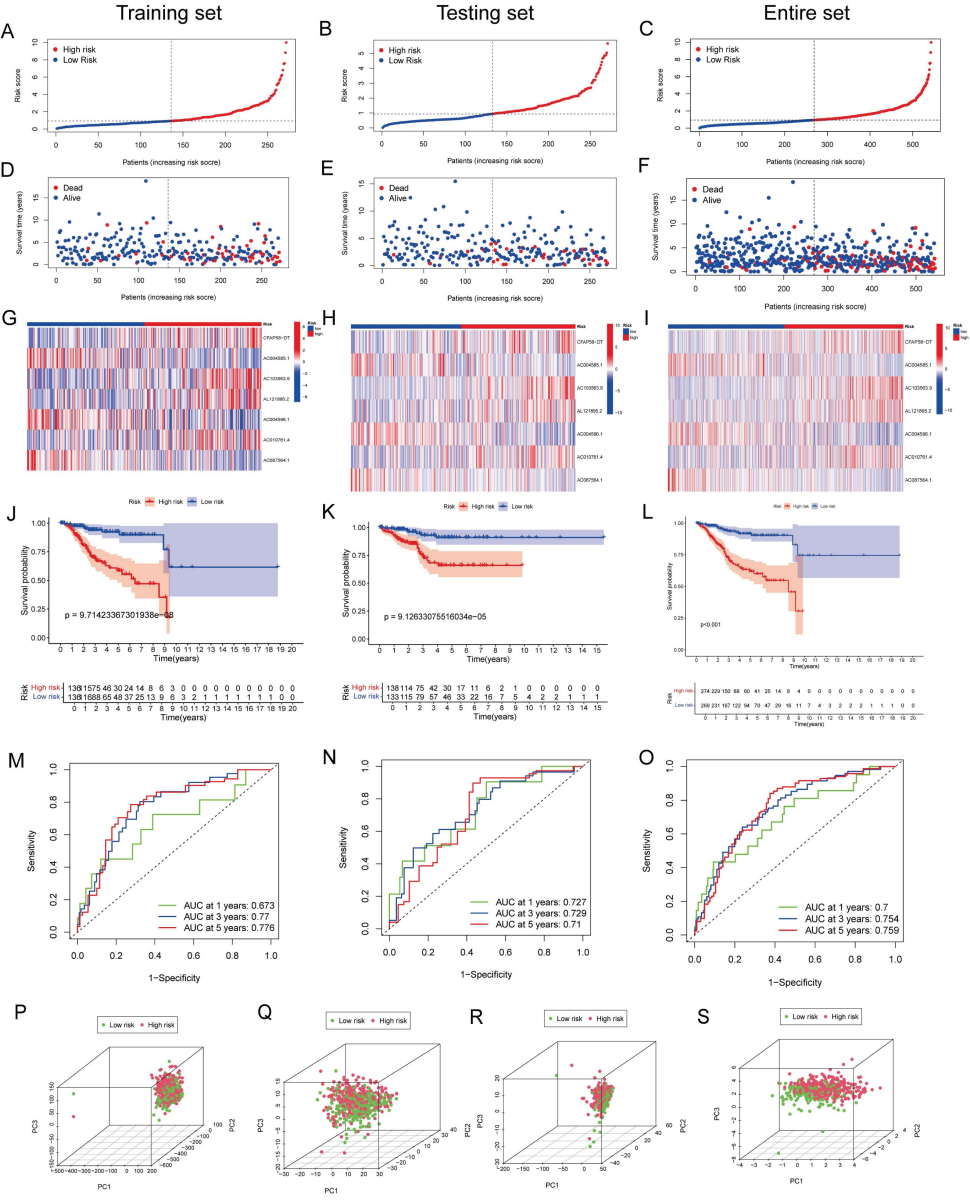
** (A-C)** Risk curves showing risk score distribution. **(D-F)** Scatter plot of survival duration and status of patients in the high-risk and low-risk groups. **(G-I)** Heatmap showing the expression levels of lncRNAs in the prognostic signature. **(J-L)** K-M survival curves for OS in the high-risk and low-risk groups. **(M-O)** ROC curves for predicting OS at 1, 2, and 3 years based on DE-ARLNCRs prognostic signature. **(P-S)** PCA based on (P) all genes, (Q) ARGs, (R) DE-ARLNCRs and (S) the 7 lncRNAs included in the signature.

Patients with EC were divided into high- and low-risk groups based on the median risk score (Figure 4A-C). In the training cohort, the high-risk group exhibited an increased mortality rate, suggesting that a higher risk score is indicative of a poorer prognosis for patients with EC (Figure 4D). The heatmap analysis (Figure 4G) revealed the differential expression of seven lncRNAs between the two groups. The K-M analysis demonstrated that the prognosis of the high-risk group was significantly worse than that of the low-risk group (P < 0.001; Figure 4J). To evaluate the accuracy of survival prediction, we plotted ROC curves, as shown in Figure 4M, with AUC values of 0.673, 0.77, and 0.776 at 1, 3, and 5 years, respectively. To further validate the applicability of the prognostic signature, the same analysis was conducted on two additional cohorts. Consistent findings were also confirmed in the testing cohort (Figure 4E, H, K and N) and the entire patient cohort (Figure 4F, I, L and O). Furthermore, we validated the grouping capability of the signature using PCA at different levels, revealing that the samples were successfully segregated into two independent groups.

### Clinical evaluation of the DE-ARLNCR prognostic signature

**Figure 5:**
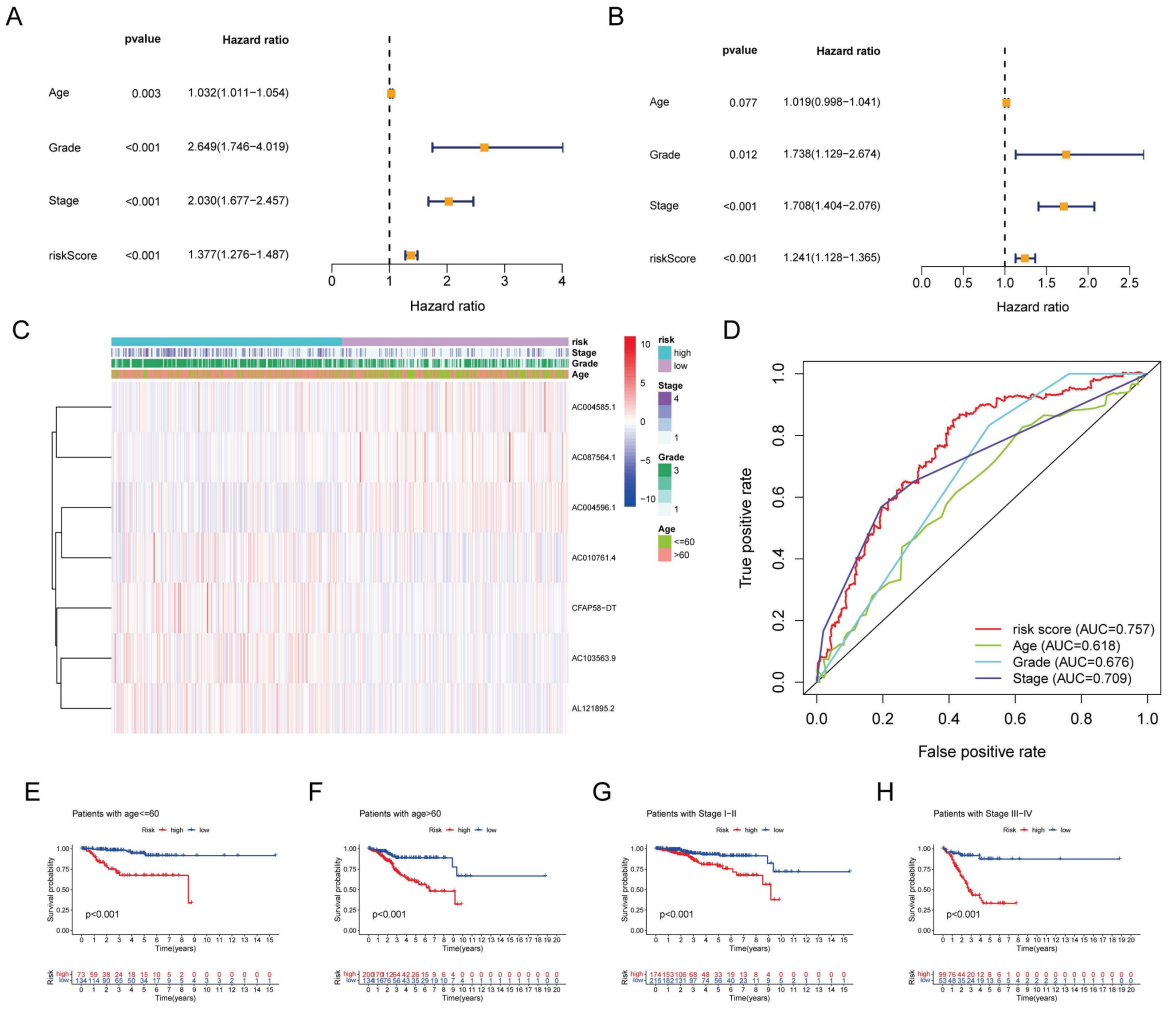
** (A)** Univariate cox regression analysis for age, grade, stage, and risk score. **(B)** Multivariate cox regression analysis for age, grade, stage, and risk score. **(C)** Distribution heatmap of 7 prognostic DE-ARLNCRs and clinicopathological variables in high-risk and low-risk groups. **(D)** ROC curves of clinical characteristics and risk score. **(E-H)** K-M survival analysis of clinical characteristics.

Univariate Cox regression analysis revealed that age, stage, grade, and risk score were significantly associated with OS in patients with EC (Figure 5A). Multivariate Cox regression analysis further demonstrated that the grade, stage, and risk score were independent predictors of OS (Figure 5B). A clinical correlation heatmap illustrated age, grade, and stage distribution across the high- and low-risk groups. The ROC curve indicated that the risk score exhibited the largest AUC (0.757) compared with age, grade, and stage. Subgroup analyses based on the stage and age of patients with EC were conducted to ascertain the predictive capability of various clinical features. It was found that the signature effectively differentiated between high-risk and low-risk groups, as evidenced by the significantly lower OS rates in the high-risk group compared to the low-risk group across the following subgroups: patients ≤60 years, patients > 60 years, patients with stage I-II, and patients with stage III-IV.

### Construction of the nomogram

**Figure 6:**
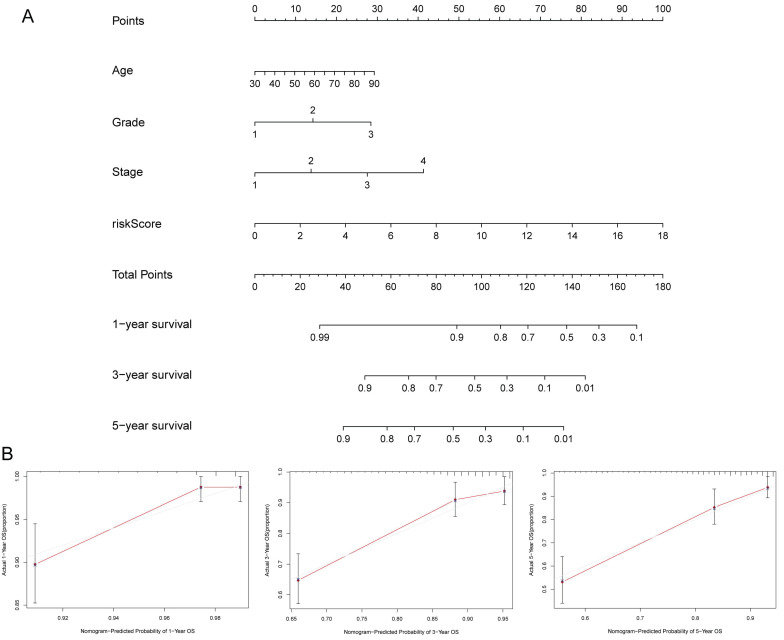
** (A)** Establishment of prognostic nomogram to predict survival of EC. **(B)** Calibration curves of the nomogram to predict 1-year and 3-year and 5-year survival.

To further predict the prognosis of patients with EC, we constructed a nomogram that included clinicopathological variables and risk scores. This nomogram predicted the 1-, 3-, and 5-year prognosis of patients with EC (Figure 6A). The calibration curves exhibited good consistency between the actual OS rates and the predicted survival rates at 1, 3, and 5 years (Figure 6B-D).

### GSEA based on the DE-ARLNCR prognostic signature

To gain further insight into the underlying molecular mechanisms between DE-ARLNCRs and EC, we performed GSEA to elucidate the possible differences in enriched signaling pathways and biological functions between the high- and low-risk groups. The results indicated that the high-risk group exhibited enrichment in pathways such as the calcium signaling pathway, cardiac muscle contraction, dilated cardiomyopathy, neuroactive ligand receptor interaction, PPAR signaling pathway, tight junctions, and type II diabetes mellitus (Figure S2A). These pathways are typically associated with intercellular interactions, cell proliferation, differentiation, apoptosis, angiogenesis, and glucose metabolism 16-18. The low-risk group demonstrated enrichment in pathways including allograft rejection, cytokine-cytokine receptor interaction, fatty acid metabolism, graft-versus-host disease, Leishmania infection, ribosomes, and type I diabetes mellitus (Figure S2B). These pathways are closely associated with immune responses, inflammatory reactions, and metabolic regulation 19-21. Furthermore, the high-risk group was enriched in functions such as cell-cell adhesion via plasma membrane adhesion molecules, synapse organization, cell body, presynapse, and cation transmembrane transporter activity (Figure S2C). The low-risk group showed enrichment in functions such as axoneme assembly, microtubule bundle formation, ciliary plasma, immunoglobulin complex, and T-cell receptor complex (Figure S2D). In summary, DE-ARLNCRs may be involved in the regulation of various cellular processes that influence EC progression, including intercellular interactions, cell proliferation, differentiation, apoptosis, angiogenesis, glucose and fatty acid metabolism, immune responses, and inflammatory regulation. These findings not only provide a new perspective for understanding the role of DE-ARLNCRs in EC but also provide a basis for further exploration of their potential as therapeutic targets. Therefore, we further explored the differences in immune function between high- and low-risk groups.

### Immune function analysis between high- and low-risk groups

**Figure 7:**
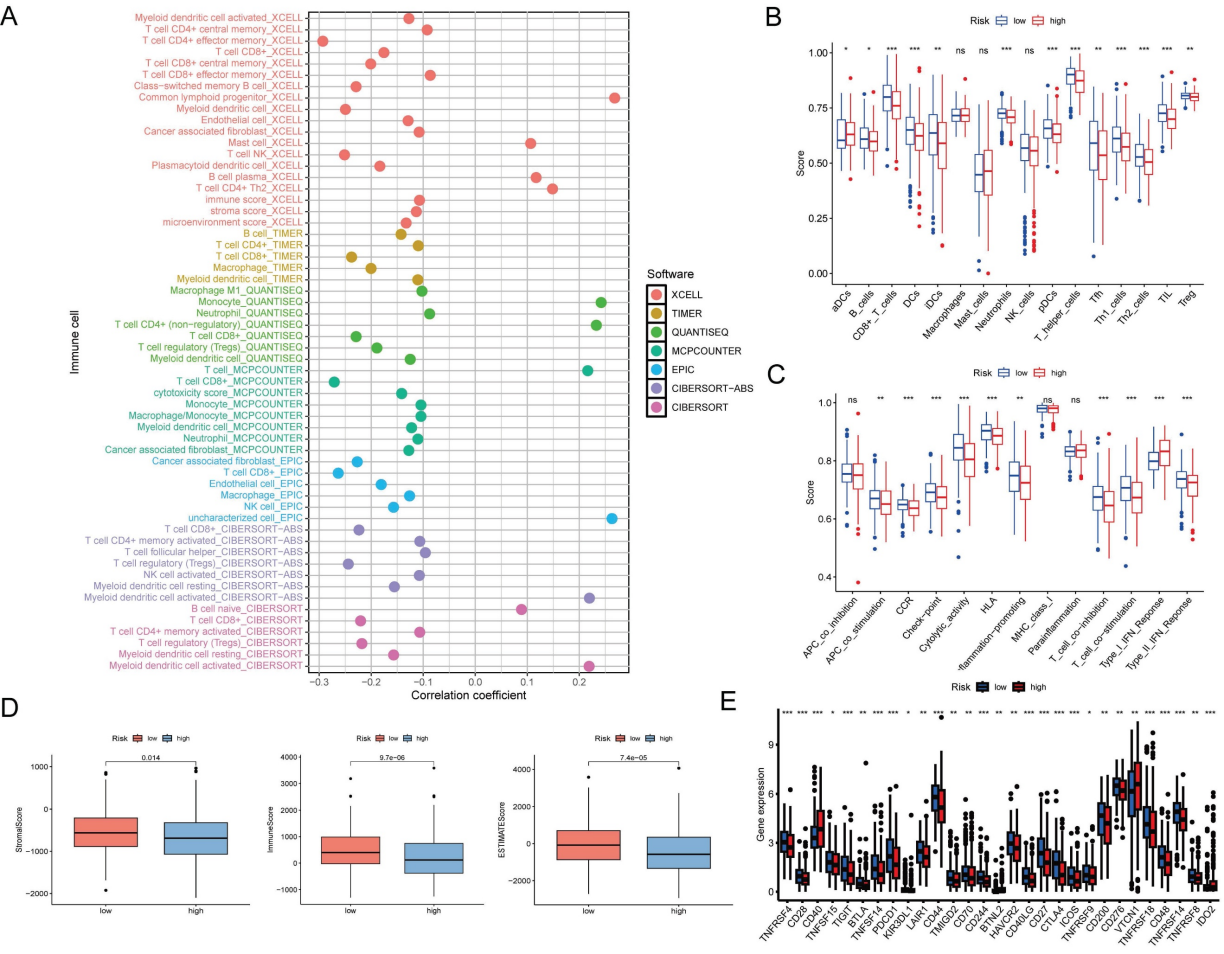
** (A)** Bubble chart showing the correlation between the immune cell array and risk scores. **(B)** Immune cell, and **(C)** immune function differences through ssGSEA. ***, P<0.001; **, P<0.01; *, P<0.05; ns, not significant. **(D)** Box plots of stromal score, immune score, and ESTIMATE score for two groups. **(E)** Immune checkpoints analysis in two groups. ***, P<0.001; **, P<0.01; *, P<0.05.

To investigate the relationship between the prognostic signature and immune-infiltrating cells, we employed seven standard methods for comprehensive integration and analysis 22. The results indicated a broad negative correlation between the immune cell array and the risk scores (Figure 7A). To further explore this, we used single-sample (ss)GSEA to quantify the enrichment scores of different immune cell subsets and immune functions. Significant differences were observed in the proportions of most immune cell types and immune functions between the two groups. Cells, such as B cells, CD8+ T cells, DCs, immature dendritic cells (iDCs), neutrophils, plasmacytoid dendritic cells (pDCs), T helper cells, T follicular helper (Tfh), T helper 1 (Th1) cells, T helper 2 (Th2) cells, tumor-infiltrating lymphocytes (TIL), and T cells regulatory (Tregs), were higher in the low-risk group, while activated dendritic cells (aDCs) showed a greater level in the high-risk group (Figure 7B).

In addition, APC co-stimulation, chemokine receptor (CCR), checkpoint, cytolytic activity, human leukocyte antigen (HLA), inflammation-promotion, T cell co-inhibition, T cell co-stimulation, and type II IFN response were more active in the low-risk group, except for the type I IFN response (Figure 7C). Furthermore, the stromal, immune, and ESTIMATE scores of low-risk patients exceeded those of high-risk patients (Figure 7D).

The advent of immune checkpoint inhibitors (ICIs) has revolutionized therapeutic approaches for a wide range of tumor types, including EC 23. In this study, we found that certain immune checkpoints were differentially expressed between the high- and low-risk groups (Figure 7E). These findings suggest that DE-ARLNCRs may play a pivotal role in modulating immune responses to tumors and that the DE-ARLNCR prognostic signature has the potential to aid in predicting the efficacy of immunotherapy in patients with EC.

### Sensitivity of anticancer drugs between high- and low-risk groups

We aimed to establish a correlation between our prognostic signature and the efficacy of drug treatments for EC. Through the analysis of commonly prescribed anticancer drugs, we found that the low-risk group exhibited greater sensitivity to most drugs, such as 5-Fluorouracil, Olaparib, Docetaxel, Paclitaxel, Topotecan, and Dabrafenib; however, their sensitivities to WEHI-539 and WIKI4 were lower than that of the high-risk group (Figure S3). This suggests that our signature has potential as a predictor of chemotherapy sensitivity and offers new options for future clinical treatment strategies.

### Expression and functional validation of *CFAP58-DT* in EC

**Figure 8:**
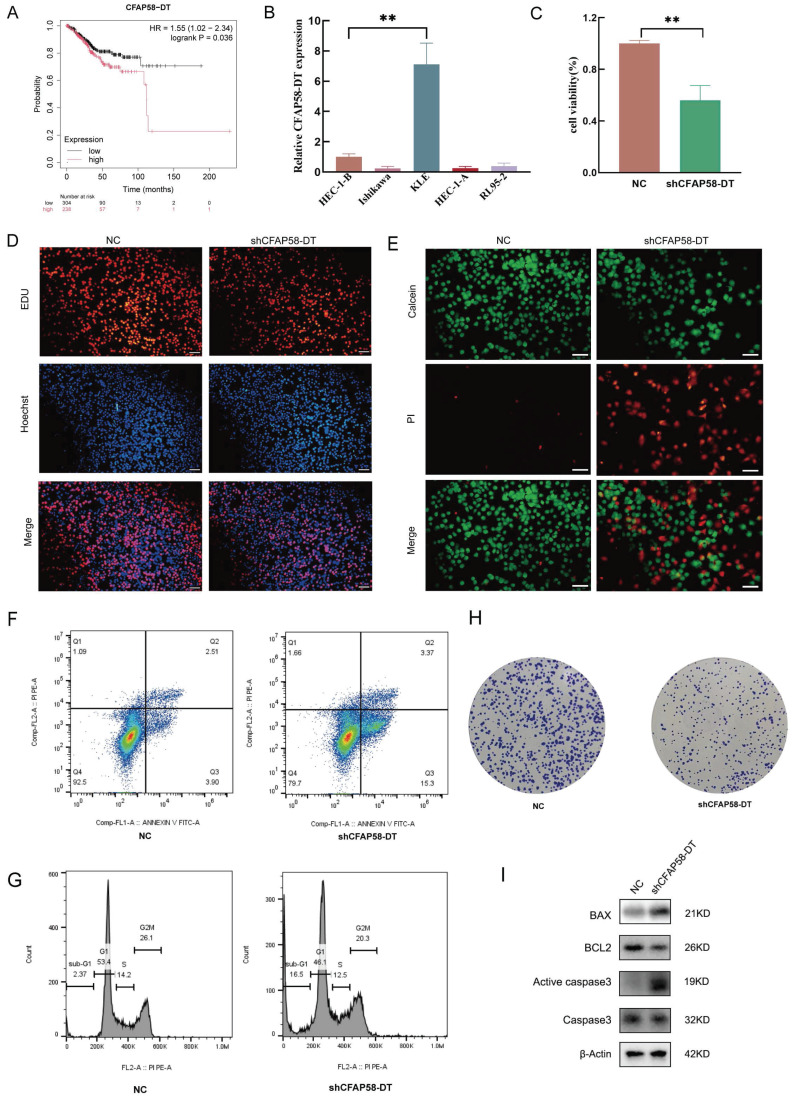
** (A)** KM Plotter database exhibited the relationship between expression of CFAP58-DT and OS in EC patients. **(B)** qRT-PCR showed the CFAP59-DT expression in different EC cells. **(C-H)** CCK8, EdU, Calcein/PI cell staining, flow cytometry and colony formation assays showed shCFAP59-DT enhanced the apoptosis and inhibited proliferation of KLE cells. **(I)** Western blot showed the protein levels of BAX, Bcl2, Caspase3, Active Caspase3.β-Actin is an internal parameter.

To examine the potential involvement of these lncRNAs in EC development, *CFAP58-DT* was selected for further studies. We first used the external database KM Plotter for verification, which corroborated our results, indicating that *CFAP58-DT* was predictive of poor prognosis (Figure 8A) 24. After evaluating the expression of *CFAP58-DT* in EC cell lines, we transfected the *shCFAP58-DT* plasmid into KLE cells for loss-of-function experiments (Figure 8B). The CCK8 assay showed that the inhibition of *CFAP58-DT* repressed the viability of KLE cells (Figure 8C). The EdU assay, calcein/PI cell staining, flow cytometry and colony formation assays showed that *CFAP58-DT* knockdown enhanced the apoptosis and inhibited proliferation of KLE cells (Figure 8D-H). The protein expression levels of BAX, BCL2, caspase3 and active caspase-3, which are key indicators of cellular apoptosis, were significantly upregulated in the *shCFAP58-DT* cells (Figure 8I). Therefore, our results indicated that *CFAP58-DT* plays a significant role in the regulation of proliferation and apoptosis.

**Figure 9:**
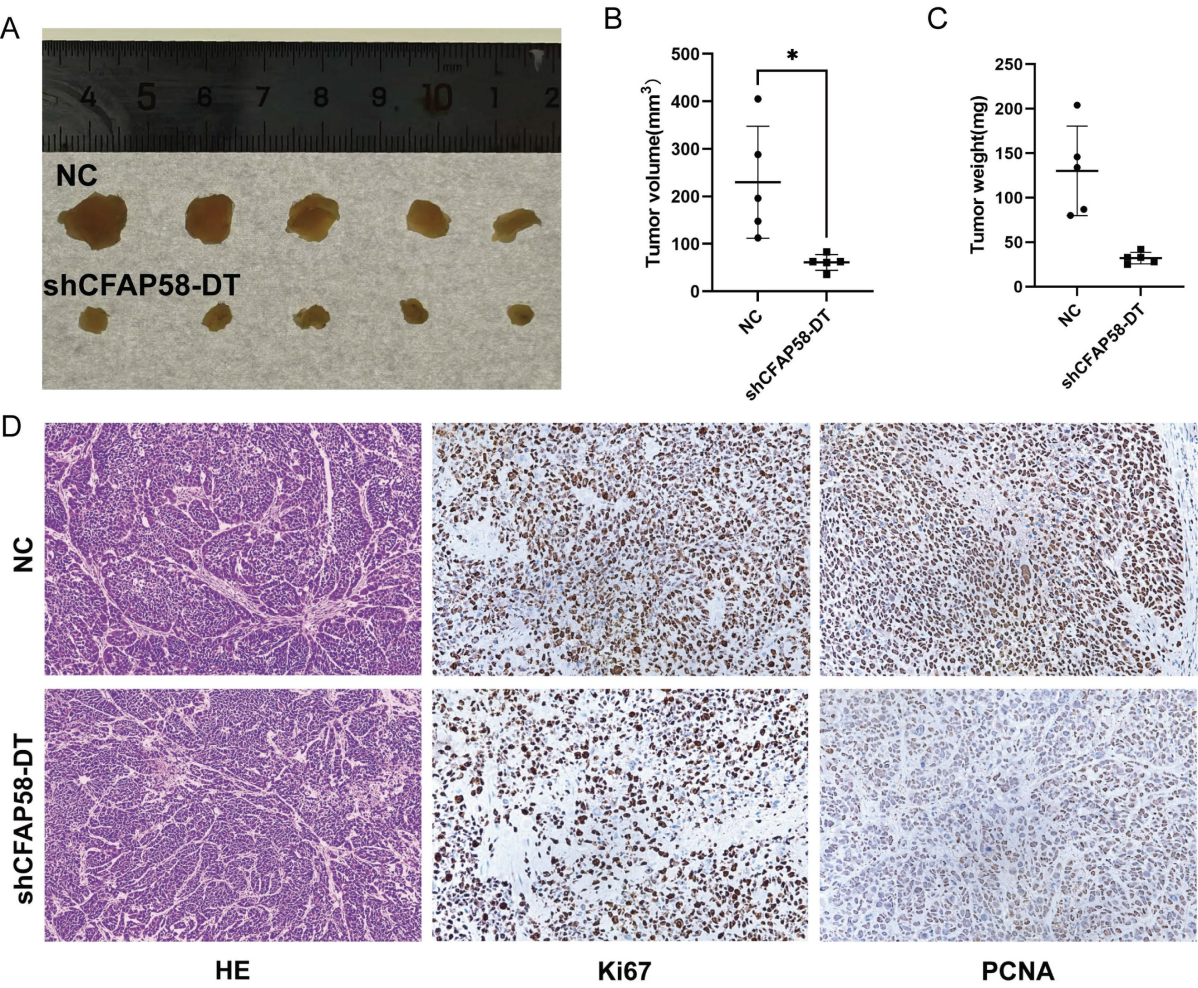
** (A)** Images of tumors by subcutaneous injection at the end point of BALB/c nude mice. **(B)** Tumor volume measured at the end point. **(C)** Tumor weight measured at the end point.** (D)** Images of HE staining and immunohistochemistry (IHC) of subcutaneous xenograft tumors.

To explore the effects of *CFAP58-DT* on EC growth *in vivo*, a nude mouse tumorigenicity assay was performed. KLE cells were stably transfected with *shCFAP58-DT* and injected subcutaneously into female BALB/c nude mice. After 4 weeks, the tumor volumes in *shCFAP58-DT* mice were significantly smaller than those in the NC group (Figure 9A-C). HE staining revealed the cellular morphology of subcutaneously transplanted tumors. In addition, immunohistochemical staining of these subcutaneous tumors demonstrated that the *CFAP58-DT* knockdown group exhibited a decrease in the expression of Ki67 and PCNA in tumor tissues (Figure 9D). Overall, the results of these experiments revealed that *CFAP58-DT* may play a crucial role in the development of EC tumors.

## Discussion

As a highly heterogeneous malignant tumor of the female genital tract, the incidence and disease-associated mortality of EC are rapidly increasing globally 25, 26. Although most patients with EC present with early-stage disease and have a favorable prognosis after surgery, the prognosis remains poor in patients with advanced-stage EC, disease recurrence, or distant metastases 27. Based on molecular characterization, TCGA Research Network has identified four widely accepted EC subtypes: POLE ultra-mutated, mismatch repair-deficient, copy number low, and copy number high 28. However, this classification cannot adequately and accurately predict patient prognosis. Therefore, it is of paramount importance to identify novel biomarkers that can effectively predict the prognosis of patients with EC, thereby providing them with more precise prognostic models and tailored treatment plans.

Anoikis, a form of programmed cell death induced by detachment from neighboring cells or the ECM, has received widespread attention since its formal nomination by Frisch and Francis in 1994 6. Multiple pathways regulate anoikis, ultimately leading to caspase activation and DNA fragmentation, resulting in cell death 8, 29. Anoikis resistance and anchorage-independency empower tumor cells to proliferate and infiltrate neighboring tissues, facilitating their dissemination throughout the body and ultimately leading to metastasis 30, 31. Overcoming anoikis is a pivotal stage in the sequence of transformations that cancer cells undergo during their progression to malignancy 32. Consequently, a deeper understanding of the mechanisms underlying anoikis resistance could aid in the inhibition of tumor progression and the prevention of metastasis.

Although once considered transcriptional noise, lncRNAs have emerged as critical regulators of gene expression and are key players in cancer biology 33-35. Promoter methylation of the lncRNA *LOC554202* leads to decreased *miR-31* expression, which contributes to breast cancer invasion and metastasis 36. In pancreatic cancer, *HOTTIP* promotes progression and gemcitabine resistance by regulating *HOXA13* 37. In many relevant studies, lncRNAs have been investigated as potential key factors in the prognostic assessment of tumors 38-40. Gaining a profound understanding of the functional roles of lncRNAs in cancer has the potential to facilitate the development of novel lncRNA-based interventional strategies, thereby opening new avenues for the treatment or prevention of cancer.

Therefore, it is necessary to conduct further research on the co-regulatory functions of anoikis and lncRNAs in EC. In this study, by integrating the transcriptomic data of patients with EC from the TCGA database and the ARGs from the GeneCards and Harmonizome database, we identified seven DE-ARLNCRs (*CFAP58-DT, AC004585.1, AC103563.9, AL121895.2, AC004596.1, AC010761.4*, and *AC087564.1*) and constructed a prognostic signature.

Of the seven DE-ARLNCRs included in the signature, *CFAP58-DT* and *AC004585.1* have been previously reported. *CFAP58-DT* has been shown to be a potential cofactor for *MDA5*; it enhances the innate immune response to viral infections by facilitating the oligomerization and activation of *MDA5* 41. Furthermore, it has been proven to be able to regulate cell viability, invasion, and migration 42. Our results also indicated that *CFAP58-DT* may be a risk indicator in patients with EC, which is related to poor prognostic outcomes.

Based on our experiments, the silencing of *CFAP58-DT* significantly affected cell proliferation, promoted cell apoptosis, suppressed the growth of subcutaneous tumors in nude mice, and reduced tumor malignancy. These data were not available in previous studies. *AC004585.1* has been reported to predict the outcomes of patients with breast and bladder cancer in several studies 43, 44.

In the validation of our signature, there is a strong correlation between the OS and the risk score in patients with EC. The AUCs for predicting 1-, 3-, and 5-year OS were 0.673, 0.77, and 0.776, respectively. PCA showed a good grouping ability of the signature. Compared to other clinical parameters, such as age, grade, and stage, the risk score had the best predictive effect, further proving that the signature is an extremely accurate predictor of patient prognosis. In addition, we established a nomogram for the personalized prediction of prognosis in patients with EC. The calibration curves proved that the nomogram had reliable prediction efficiency, showing good consistency between prediction and actual survival.

GSEA revealed differences in the enrichment of signaling pathways and biological functions between the high- and low-risk groups. The high-risk group exhibited enrichment in multiple signaling pathways related to cellular function and metabolism. In contrast, the low-risk group showed enrichment of pathways related to immunity and infection. Regarding biological functions, the functional enrichment of the high-risk group was primarily focused on intercellular communication and material transport. The low-risk group showed enrichment in functions related to cellular structural stability and regulation of immune responses. These findings indicate that DE-ARLNCRs may play a role in regulating diverse cellular processes that impact the progression of EC, including intercellular interactions, cell proliferation, differentiation, apoptosis, angiogenesis, glucose and fatty acid metabolism, immune responses, and inflammatory regulation.

The immune microenvironment in cancer represents a complex and pivotal ecosystem that significantly influences tumor initiation, development, and progression 45, 46. Through an integrated analysis of multiple recognized and effective methods, the results indicated that patients in the high-risk group typically exhibited lower levels of immune cell infiltration, suggesting a suppressed immune system that was unable to effectively control and monitor tumor development. Furthermore, high-risk patients demonstrated a lower stromal score, immune score, and ESTIMATE score compared to low-risk patients, reflecting a tumor microenvironment that was less conducive to immune cell infiltration and functionality, resulting in a so-called “cold immunity” state. Specifically, within the high-risk group, only the type I IFN response among key immune-related functional pathways was more active than that in the low-risk group. However, IFN-I signaling has been reported to lead to more aggressive phenotypes, not only limiting the expansion of effector cells but also inhibiting their functional adaptability, thereby contributing to poor immunogenicity and clinical outcomes 47. Notably, there were significant differences in the expression of certain immune checkpoints between the high- and low-risk groups. This finding implies that DE-ARLNCRs participate in regulating the tumor immune response and aid in predicting the response of patients with EC to immunotherapy. With the widespread application of ICIs across various tumor types, this finding offers potential targets for immunotherapy in EC.

This study has certain limitations. One major limitation is that our prognostic signature was developed using publicly available transcriptomic and clinical data from TCGA, and its accuracy was evaluated via an internal testing set. Future studies should consider incorporating external validation using data from other sources or independent cohorts to achieve more comprehensive conclusions. Second, although preliminary findings have been established, the upstream and downstream regulatory mechanisms underlying the function of CFAP58-DT remain insufficiently explored and should be addressed in future investigations.

## Conclusion

Our study identified seven DE-ARLNCRs, successfully established a prognostic signature for patients with EC, and validated its robust predictive capabilities. After conducting thorough discussions on its relationship with clinical characteristics, immunity, and drug sensitivity, we found that the signature could provide effective guidance for the prognosis and treatment of patients with EC. Additionally, we verified that *CFAP58-DT* affected EC progression both *in vitro* and *in vivo*, further confirming our results. In clinical practice, there is an emphasis on planned and rational comprehensive treatment with a strong focus on individualized therapy in EC. We hope that our study will enhance the evaluation of the prognosis, molecular characteristics, and therapeutic approaches in patients with EC, potentially paving the way for future clinical applications and translation.

## Supplementary Material

Supplementary figures and tables.

## Figures and Tables

**Table 1:** The clinical characteristics of patients in different cohorts.

Variables	Type	Training cohort (n = 272) (%)	Testing cohort (n = 271) (%)	Entire TCGA dataset (n = 543) (%)
Age	≤60	108 (39.7)	99 (36.5)	207 (38.1)
	>60	164 (60.3)	170 (62.7)	334 (61.5)
	Unknown	0	2 (0.7)	2 (0.4)
Grade	Grade 1	48 (17.6)	51 (18.8)	99 (18.2)
	Grade 2	56 (20.6)	65 (24.0)	121 (22.3)
	Grade 3	168 (61.8)	155 (57.2)	323 (59.5)
Stage	Stage I	159 (58.5)	180 (66.4)	339 (62.4)
	Stage II	26 (9.6)	26 (9.6)	52 (9.6)
	Stage III	68 (25)	55 (20.3)	123 (22.7)
	Stage IV	19 (7.0)	10 (3.7)	29 (5.3)

**Table 2:** Multivariate cox regression analysis of DE-ARLNCRs.

id	coef	HR	HR.95L	HR.95H	pvalue
CFAP58-DT	0.506634	1.659696	1.015894	2.711494	0.04308
AC004585.1	-0.3563	0.700261	0.455276	1.077072	0.104809
AC103563.9	1.066652	2.905636	1.069599	7.893348	0.036446
AL121895.2	0.311126	1.364961	0.927871	2.00795	0.114146
AC004596.1	-0.74335	0.475516	0.292538	0.772945	0.002708
AC010761.4	0.376952	1.457835	1.046708	2.030445	0.025746
AC087564.1	-1.74644	0.174394	0.030023	1.013001	0.051706
