# Supplementary material for: Identification of an Anoikis-associated LncRNA Signature to Predict the Clinical Prognosis and Immune Function of Patients with Endometrial Cancer
Source: Int J Med Sci. 2025 May 7;22(10):2502–17. doi: 10.7150/ijms.107243 (PMC12080581; doi:10.7150/ijms.107243)
Supplement: Supplementary file 1 — Supplementary figures and tables. [file ijmsv22p2502s1.pdf]

Supplementary

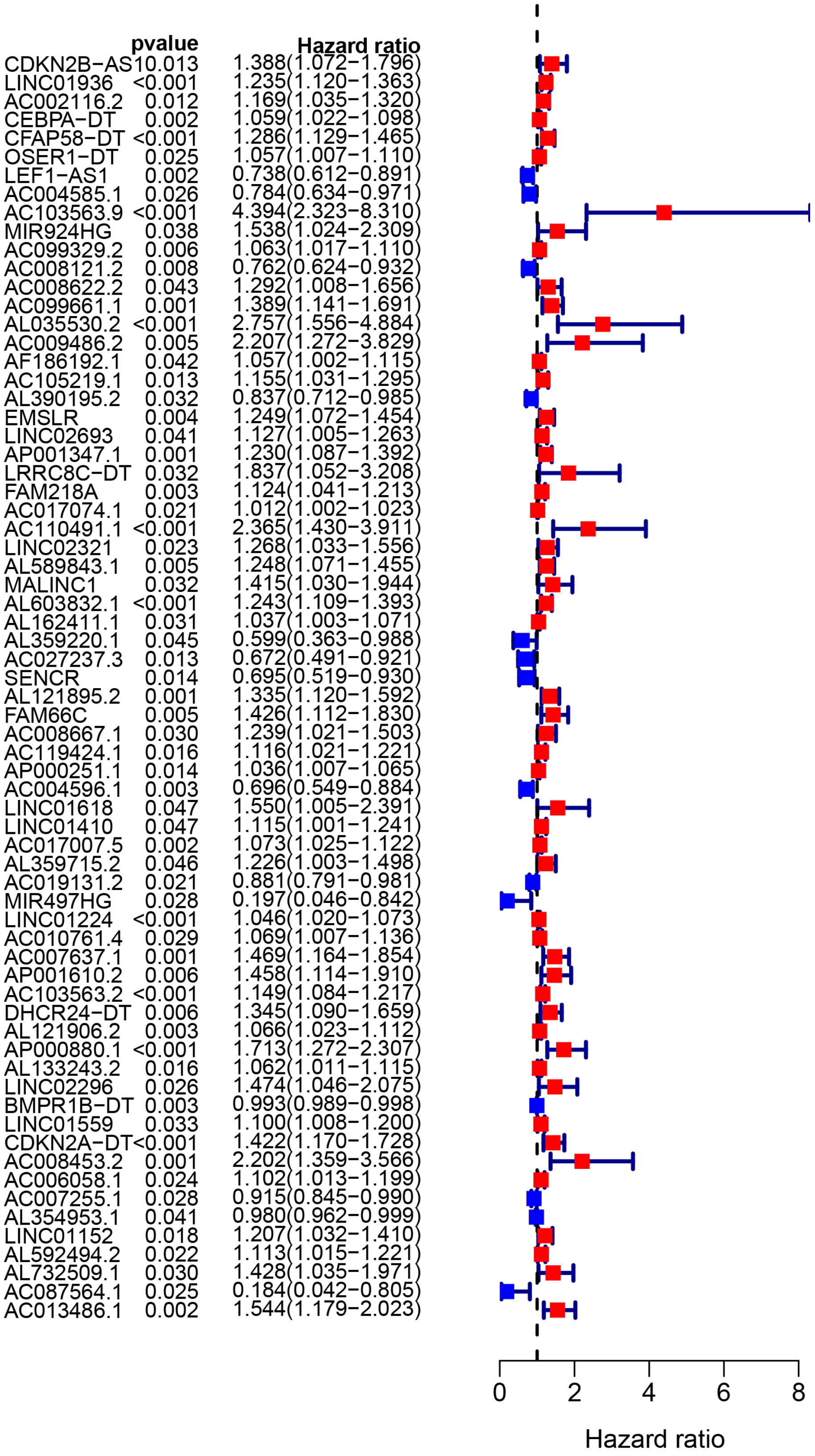

Figure S1 univariate cox regression analysis of 68 DE-ARLNCRs

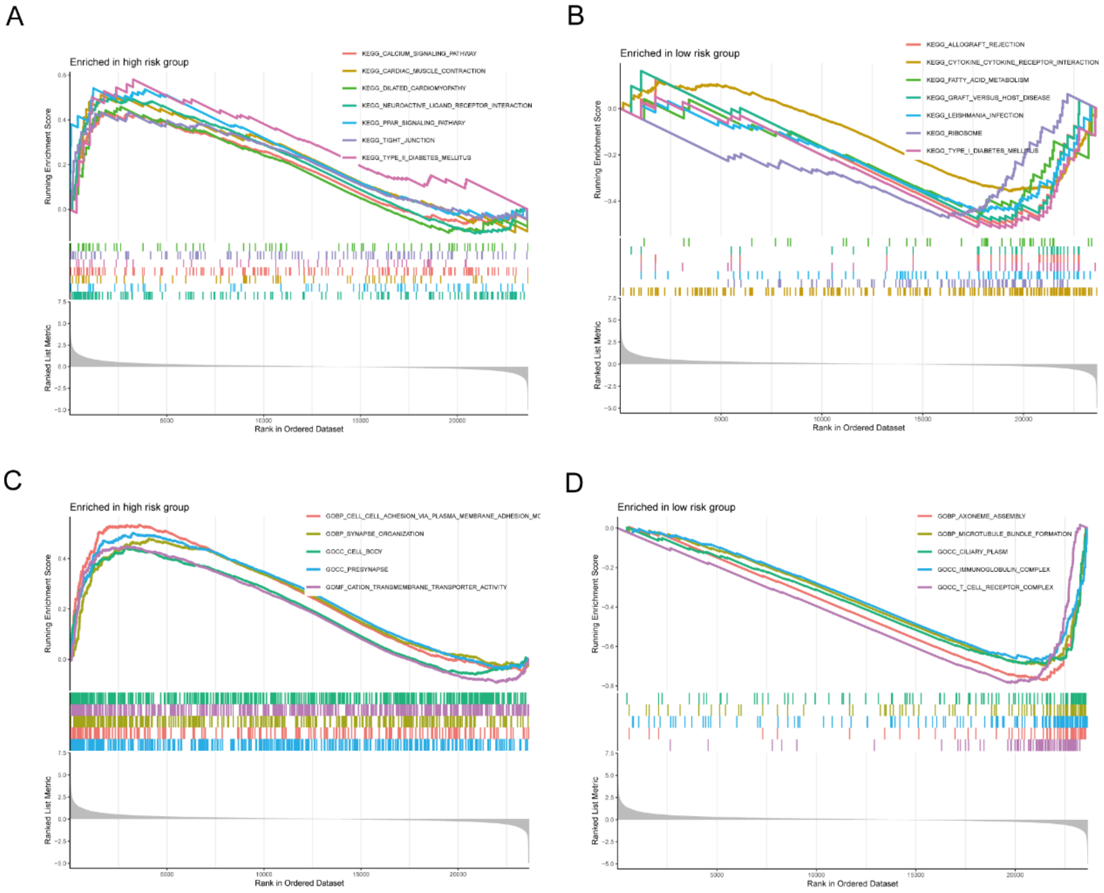

FigureS2 GSEA shows the top enriched pathways and biological functions of the high- and low-risk groups.

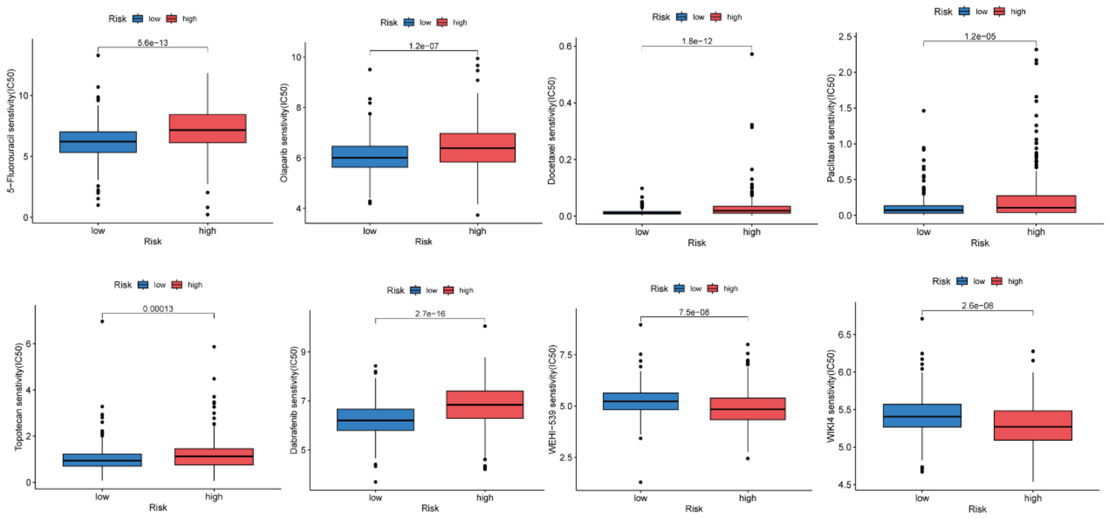

FigureS3 Estimated drug sensitivity in patients of the high- and low-risk groups. IC50 means half inhibitory centration.

cell line	PTEN	TP53	PIK3CA	ARID1A	CTNNB1	KRAS
HEC1B	/	SNV	SNV	SNV	/	SNV
Ishikawa	deletion	SNV	/	deletion	/	/
KLE	/	SNV	/	/	/	/
HEC1A	/	SNV	SNV	SNV	SNV	SNV
RL952	SNV	deletion	/	SNV	/	/

“/” means wild type, “SNV” means single nucleotide variant.

TableS1 The mutation statuses of six driver genes across the individual cell lines used from the DepMap portal website.
